# ASW-YOLO: Lightweight Gear Defect Detection with Improved YOLOv8n

**DOI:** 10.3390/ma19112309

**Published:** 2026-05-29

**Authors:** Zhecheng Luo, Bin Zheng

**Affiliations:** School of Intelligent Manufacturing, Panzhihua University, Panzhihua 617000, China

**Keywords:** gear, YOLO, lightweight, feature enhancement, loss function, attention mechanism

## Abstract

Aiming at the problems of diverse defect types, large-scale differences, and complex background interference in gear surface defect detection, a lightweight model, ASW-YOLO, is proposed based on YOLOv8n. By using an ADown dual downsampling module to compress feature map resolution and preserve fine-grained information. C2f_SE channel attention is introduced to enhance small-scale defect response. The CIoU is replaced with WIoU to optimize multi-scale target localization accuracy. The experiments are conducted on the gear dataset. The comparative experiments show that mAP@0.5 of ASW-YOLO reached 94.8%, an increase of 4.5% compared to YOLOv8n, with a reduction of 9.3% in parameter count and 8.5% in computational complexity. The ablation experiments confirm the effectiveness of the three modules. ASW-YOLO achieves a 4.5% increase in mAP@0.5 and a 6.1% increase in recall compared to YOLOv8n. The generalization experiments demonstrate that the mAP@0.5 fluctuation of ASW-YOLO remains below 2% under strong highlight and striped shadow. Moreover, the model maintains over 85% mAP@0.5 under motion blur. ASW-YOLO balances precision and lightweight, making it suitable for real-time quality monitoring in industry.

## 1. Introduction

In key fields such as the automotive industry, aerospace, wind power generation, and heavy-duty machine tools, gear transmission systems play a core role in motion conversion. With the continuous development of modern equipment toward heavy load and high precision, gears are subjected to complex service conditions such as alternating loads. The surfaces are prone to damage and defects. In addition, the advancement of precision manufacturing technology has put forward higher requirements for gear machining accuracy, and small-scale surface defects may also affect the operational stability of the transmission system. Therefore, an efficient and accurate method for detecting gear defects has an important engineering value in ensuring the operational efficiency of mechanical systems.

The conventional gear defect detection methods rely on manual visual inspection, contact measurement, and nondestructive testing techniques. Manual visual inspection relies on inspectors directly observing the surface of gears, which has low detection efficiency. Long-term work can cause visual fatigue, and the detection results are affected by subjective experience factors. The contact measurement methods, such as micrometers, calipers, etc., are mainly used to detect geometric dimensional deviations of gears and cannot identify surface microdefects. The conventional nondestructive testing techniques, such as magnetic particle inspection and penetrant testing, can detect surface cracks, but the testing process is complex, the efficiency is limited, and it is difficult to achieve fully automated online testing. The traditional methods mentioned above have obvious limitations in terms of detection efficiency, objectivity, and automation level, making it difficult to meet the requirement for full inspection of gear quality.

In recent years, the two-stage detection model represented by Faster R-CNN generates candidate regions through region suggestion networks, which have high positioning accuracy. However, the model structure is complex, the parameter quantity is large, and the inference speed is difficult to meet real-time detection requirements. The one-stage detection model represented by SSD and YOLO series has significant advantages in detection speed. The gear surface defects include various types, such as rust, wear, cracks, and root fractures, and there are significant differences in scale, shape, texture, and contrast among these types of defects. In addition, there may be multiple types of defects on the same gear, and the characteristics between defects interfere with each other, further increasing the difficulty of the detection model’s discrimination. The complexity of data processing and analysis is high. How to quickly locate defect areas, accurately classify defect types, and suppress the interference of complex background noise from high-resolution images with limited computing resources is a key technical challenge for achieving reliable detection.

In response to the above issues, the YOLOv8n is used as the baseline model, which has been improved in three dimensions: network structure, attention mechanism, and loss function. Thus, an improved YOLOv8n model suitable for gear defect detection has been proposed. The main improvement work includes the following four aspects.

(1) Optimization of the downsampling module. The standard convolutional downsampling module in the original backbone network is replaced with the ADown module. The ADown module combines average pooling and maximum pooling features. This module adopts a dual-path convolution design. While reducing the resolution of the feature map, it preserves fine-grained spatial information. Therefore, higher feature inputs are provided for subsequent detection layers.
(2) Introduction of attention mechanism. The original C2f is replaced with the C2f_SE, which incorporates the SE channel attention mechanism while maintaining a lightweight structure. By adjusting the weights of different channel features, the model can focus on key features related to defects, suppress background noise interference, and improve its responsiveness to small-scale defects in complex backgrounds.
(3) Improvement of the loss function. The original CIoU loss function is replaced with the WIoU. The WIoU adjusts regression weights based on the target scale. By balancing the contributions of both large and small targets during the training process, the model’s localization accuracy and recall rate on multi-scale defects can be improved.
(4) Comparative experimental verification. After completing the above improvements, the improved YOLOv8n model will be compared with the original YOLOv8n and other object detection models to evaluate their accuracy, speed, and computational efficiency performance.

The sections of this article are arranged as follows. Section 2 provides relevant research work and summarizes the current application status and limitations of existing object detection models in the field of industrial defect detection. Section 3 provides a detailed introduction to the improvement strategies of the YOLOv8n model, including optimization of the downsampling module, introduction of the attention mechanism, and improvement of the loss function. Section 4 presents the construction of experimental datasets, the setting of training parameters, and experiment results. Section 5 summarizes the research work and future research directions.

## 2. Related Works

### 2.1. Application of Deep Learning in Object Detection

Tan Mingxing [1] proposed an efficient structural design for object detection models. By constructing the Bidirectional Feature Pyramid Network (BiFPN), the multi-scale feature fusion process was optimized. At the same time, a composite scaling method is introduced to adjust each module of the network uniformly. Based on these optimizations and the EfficientNet backbone network, a new series of object detectors called EfficientDet has been developed, which achieves much higher efficiency than existing technologies under extensive resource constraints. Han Kai [2] proposed a ghost module for generating more feature maps. A series of low-cost linear transformations was applied to generate ghost feature maps to reveal information about intrinsic features. By designing a Channel-Aware Aggregation (CAA) module to enhance small target features and using a Re-parameterization Asymptotic Feature Pyramid Network (RAFPN) to optimize feature interaction, Bian [3] proposed a CRD-YOLO algorithm for component defect detection. This algorithm designs a Channel-Aware Aggregation (CAA) module based on YOLOv5. The CAA module enhances small target features, and the algorithm uses a Re-parameterization Asymptotic Feature Pyramid Network (RAFPN) to optimize feature interaction.

Zheng Danyang [4] proposed a defect detection method for rail surface and fasteners based on a deep convolutional neural network. Wang Yin [5] replaced C2f with C2f WTConv to expand the receptive field and extract features of a small target. Wang Guiqiang [6] proposed an edge-enhanced backbone network to solve the difficulty of extracting subtle features in industrial defect detection. This structure consists of an Edge-sensitive (EdgeS) module, C3E2 module, and MSDC module, aimed at enhancing the extraction and transmission of edge features. Ye Guanting [7] improved the YOLOv7 network by introducing three self-developed modules. The Swin Transformer module is used to enhance global feature modeling capability, and residual connections are incorporated to optimize gradient flow. The network is trained on a multi-scale, multi-noise dataset to enhance its robustness in detecting cracks of different sizes. Alessio Cascino [8] has developed a 3D vision system for railway freight car monitoring. The system adopts a normalized cross-correlation tracking algorithm to achieve accurate measurement of parameters such as coupler angle offset.

### 2.2. Attention Mechanism (AM) in Object Detection

Yu Changdong [9] proposed the YOLO-MRS method, which was based on the YOLOv8 model. The Multi-scale Cross-axis Attention (MCA) mechanism is introduced to establish long-distance dependencies between pixels to extract global features. The simplified spatial pyramid pooling-fast module is added to improve calculation accuracy, and ordinary convolutional layers are replaced with Refocused Convolutional (RefConv). Su Binyi [10] proposed a Residual Channel Attention Gate Network (RCAG-Net). A novel RCAG module was used to achieve multi-scale feature fusion, complex background suppression, and defect feature highlighting. Liu Linjun [11] added a Coordinate Attention (CA) module between the backbone and neck networks, which considers channel and location information and enhances the feature extraction capability of the network model. Zhu Jiahao [12] introduced a cross-space multi-scale attention after C2Dense to perform pixel-level attention on the extracted features, enhancing useful features for defect detection while suppressing background information.

Gao Yihong [13] integrated Partial Convolution (PConv) with Spatial Attention (SA) to optimize feature extraction. PConv can reduce redundant calculations, while SA can enhance key region features. It is designed to prevent features from disappearing in shallow layers while controlling computational complexity. This mechanism reduces the computational requirements on edge devices. Zhou Qiqi [14] proposed the CABF-YOLO model for detecting surface defects on steel strips. A Triple Convolutional Coordinate Attention (TCCA) module was used to extract cross-channel features for defect localization through decomposition pooling operations. Li Fei [15] proposed a Multi-Scale Coupled Attention (MSCA), which was the evolution of self-attention in object detection. Its core was to collaboratively model multi-scale information through parallel channels and spatial dual paths. It can adaptively filter and fuse multi-scale features from both semantic and spatial dimensions to solve the detection difficulty of the dispersed distribution of multi-scale targets in complex scenes. Han Tianxin [16] proposed a lightweight adaptive downsampling technique, the Intelligent Feature Concentration (IFC) module. It adaptively generates and normalizes spatial attention maps to guide the feature downsampling process. The model dynamically focuses on key regions during feature compression, effectively suppressing redundant information. This enabled it to prioritize retaining features critical for defect identification while reducing computational load.

Zhang Yongjie [17] proposed Locally Excitatory Globally Inhibitory Oscillator Region Attention Mechanism (LEGIO-RAM). It achieved intelligent focusing of fault feature images within the object detection framework through dynamic interaction between local excitation enhancement and global inhibition feedback. Diana Novak [18] adopted a Coordinate Attention (CA) mechanism to solve the challenges of underground detection. This mechanism encoded feature maps along the horizontal and vertical directions of spatial coordinates. It can effectively locate key body parts of miners under conditions of occlusion or uneven lighting. Ma Tianci [19] integrated CA into the neck network of the YOLO. By encoding the spatial coordinate direction of the feature map, the positional information was embedded into the channel attention weights. This method enhanced the position sensitivity in the feature map, helping to distinguish between defect hot spots and background noise, and improved the recognition ability of surface defects under complex structures. Cai Yijie [20] introduced Shuffle Attention (SA) based on YOLO. This mechanism grouped the channels of the feature map and spatial attention in parallel within each group to facilitate cross-group feature interaction. Yang Yiming [21] proposed a GMG-LDefmamba-YOLO model that integrates two core modules. The Gaussian mask gear convolution module suppressed background noise by dynamically generating weight maps. The linear deformable Mamba module combined the local sampling capability of deformable convolutions with the global sequence modeling of state space models.

### 2.3. Loss Function in Object Detection

Liu Can [22] proposed a Powerful-IoU loss function that combined an adaptive penalty factor for object size and a gradient adjustment function based on anchor box quality. The PIoU loss guided anchor boxes to regress along effective paths, thus converging faster than existing IoU-based losses. Zhang Yifan [23] introduced an Effective Intersection over Union (EIOU) loss, which explicitly measures the differences in three geometric factors, namely, overlap area, center point, and side length in Bounding Box Regression (BBR). A focal loss regression version was also proposed to focus the regression process on high-quality anchor boxes. Combining the above two parts, the Focal-EIOU loss was obtained. Cheng Dong [24] proposed Controlled Distance IoU (CDIoU) and Controlled Distance IoU loss functions (CDIoU loss), which enhanced several classic and emerging models without increasing module parameters. Sun Yong [25] introduced a new Fused IoU (FIoU) loss function, which achieves excellent performance.

Xu Xiaowo [26] proposed a Distance Penalty IoU Loss (DPL) by introducing a distance penalty term into the traditional IoU calculation. It suppressed redundant prediction boxes with low localization quality. The loss function, by penalizing distance deviations, made the network pay more attention to samples with accurate localization during training. Xiong Chenqin [27] adopted the Minimum Point Distance (MPD) as the loss function and optimized the matching accuracy of crack geometric features. It replaced the traditional IoU calculation method, allowing the model to focus more on pixel-level alignment of crack edges during training. Ding Shen [28] combined Efficient IoU with Focal-F1 Loss to obtain the Focal-EIoU loss function. EIoU optimized bounding box regression, calculated through the distance between center points and the difference in width and height. Focal-F1 Loss adjusts the weights of positive and negative samples to reduce the contribution of easily classifiable samples to the total loss. Ni Xuefeng proposed an attention neural network based on IoU guided center-point estimation [29]. Additionally, the IoU decoupling and task alignment strategies were introduced to reduce the coupling interference between classification and regression tasks, balancing the learning process [30]. Pei Shi replaced the CIoU with the WIoU loss function in DVCW-YOLO for PCB defect detection, improving generalization and small-object localization, achieving 99.3% mAP@0.5 [31].

### 2.4. Advances in Gear Defect Detection Technology

Zhang Dehai [32] reviewed the development history of gear defect detection technology. The current technologies face challenges such as multi-modal data fusion, real-time computational resource constraints, and small sample generalization. The solutions included utilizing Transformers for cross-modal feature alignment, employing lightweight models to reduce parameters, and applying contrastive learning to enhance accuracy in small sample scenarios. Yuan Haibing [33] incorporated an efficient architecture of spatial pyramid pooling layers into the backbone network to enhance the model’s ability to detect irregular defects. In the neck network, the BiFormer attention mechanism was implemented to improve the detection performance of small-scale defects. Liu Yifan [34] collected images through a multi-source system and established the dataset containing 7038 defect instances. The sliding windows and global non-maximum suppression were employed to handle small and medium-sized defects. The DDPM-ASTU-Net generation model was used to synthesize samples to balance the data. Zhang Bufan [35] proposed the OGD-YOLO method for detecting surface defects on gearbox gears. This method utilized the same scale cross-layer feature connections to improve the feature concatenation module to suppress irrelevant information. The VoV-GSCSP module was used to construct a lightweight neck, reducing computational complexity. Based on the analysis of image filtering technology, Yu Liya [36] developed a background weakening algorithm for gear microdefect detection. The proposed S-YOLO model was utilized for online gear defect detection, exhibiting enhanced recognition capabilities for microdefects in complex backgrounds and demonstrating robustness in the algorithm.

Yan Rui [37] adopted the ShuffleNetv2 module as the backbone to reduce the number of gigaflops per second and the number of parameters. The transposed convolution upsampling was employed to enhance the learning capability of the network. During the prediction, SIoU_Loss was selected as the bounding box regression loss function to accelerate convergence speed. Zhou Xin [38] proposed a U-shaped spatial attention Transformer model for gear tooth surface defect detection. The method employed a window-based mechanism to construct a pyramid receptive field in the single self-attention layer, capturing both fine-grained and coarse-grained features simultaneously to achieve multi-scale information fusion. Shi Zhaoyao [39] proposed the plastic gear defect (PGD-net) detection network to address various surface defects that occur after the injection molding of plastic gears. It utilized an improved Focal-IoU loss function to solve data imbalance issues and introduced a CoordConv layer to enhance generalization capabilities. Wang Siyu [40] proposed a machine vision-based detection method for pitting defects on tooth surfaces. The U-net network was employed for defect detection and segmentation of spur gears.

Despite significant progress in the aforementioned research, gear surface defect detection still faces the following limitations. The accuracy of multi-scale defect recognition is not high. The ability to resist interference is weak, and the interference caused by the complexity of gear defect types and high-frequency noise leads to a high false detection rate. There is a contradiction between detection accuracy and model lightweight, as lightweight models are prone to missing small-sized targets. This paper aims to address the aforementioned issues and make improvements.

## 3. Methods

### 3.1. YOLOv8n Framework Structure

YOLOv8n is a computer vision model launched by Ultralytics. YOLOv8n introduces the improved CSP framework with the two convolutions (C2f) module in the backbone network, which serves as a fundamental component for efficient feature extraction. By optimizing the CSP structure, it reduces redundant computations while enhancing gradient flow. It combines the lightweight design with a multi-scale feature fusion strategy, effectively enhancing the model’s inference speed and target localization accuracy. Furthermore, YOLOv8 innovatively utilizes the PAN in the neck network. As a core technology for enhancing feature interaction, this structure establishes efficient information paths between different-scale features, achieving bidirectional fusion of shallow-level detail information. It enhances the model’s ability to detect multi-scale targets in complex backgrounds. The detection head of YOLOv8n achieves a balance between detection performance and inference efficiency through structural re-parameterization and hybrid convolution optimization.

### 3.2. Key Innovation Module

#### 3.2.1. ADown Module

**Figure 1:**
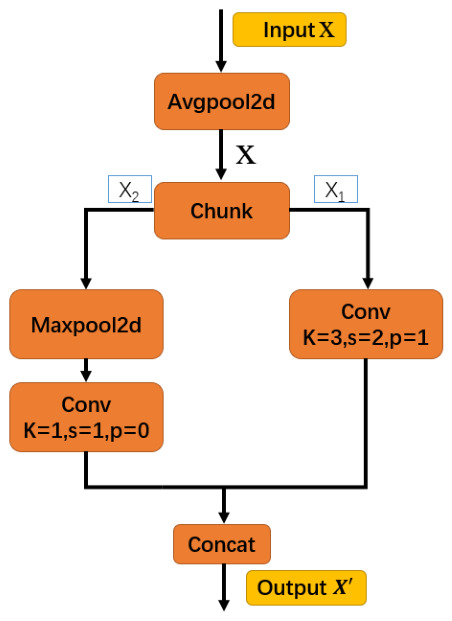
The ADown structure diagram.

Through downsampling, irrelevant detailed information related to gear defects can be removed, highlighting key features in the image. This paper draws inspiration from the ADown module introduced in YOLOv9 and uses it to replace some traditional downsampling modules. By substituting this module for some standard convolution modules in the original network structure, the computational load of the model is significantly reduced [41]. The calculation process is shown in Figure 1.

As can be seen in Figure 1, the input X of ADown first passes through an average pooling layer to retain the global information of spatial features to the greatest extent. Then, the feature map is divided into two branches, reducing the spatial feature size of the gear defect image. The first branch of ADown downsampling adopts a 3 × 3 convolution to extract the feature information of the gear defect image. The second branch first uses max pooling to reduce the spatial feature size further, enhancing the ability of the backbone network to capture important local features of the defect image. Then, it uses a 1 × 1 convolution to extract the spatial features of the defect image. These two pooling operations achieve the extraction of feature images at different scales and concatenate the spatial features at different scales. This method preserves the main feature information of the gear defects and reduces the computational and parameter count of the model. Therefore, the running speed of the improved model is improved.

#### 3.2.2. C2f_SE Module Incorporating the Squeeze-Excitation Attention Mechanism (SEAM)

The AM mimics the human visual and cognitive systems. By introducing AM, the networks can improve the performance and generalization ability of the model [42]. The SEAM is a classic channel attention module. It compresses the feature map into channel-level descriptors, then utilizes a fully connected layer to construct dependencies between channels and generate weights, and finally, it recalibrates the original features through channel-wise multiplication. This mechanism achieves adaptive adjustment of the importance of feature channels. It enhances the model’s ability to extract important semantic information. To enhance the representational power, the lightweight SEAM is integrated into C2f. It enables the network to have the function of dynamic channel feature recalibration.

**Figure 2:**
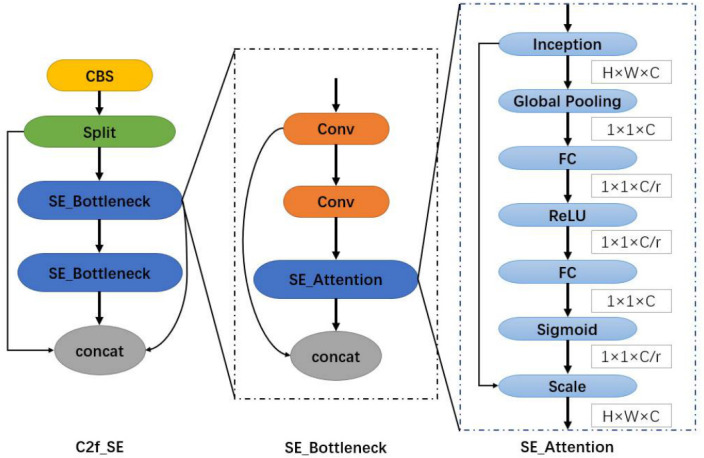
The structure diagram of the C2f_SE module.

The network structure of C2f after integrating the SEAM is shown in Figure 2. Firstly, the residual module (Bottleneck) of C2f undergoes two convolution operations, integrates the SE attention mechanism, and then performs concatenation to obtain SE_Bottleneck. Subsequently, multiple SE_Bottleneck modules are fully connected to obtain the improved C2f_SE.

**Figure 3:**
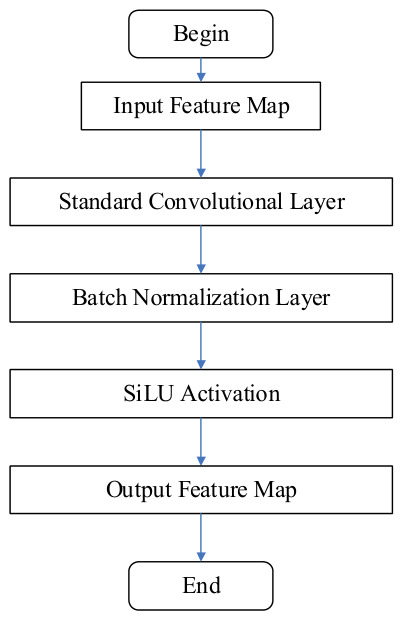
The structure diagram of the CBS module.

The CBS module is the fundamental module consisting of convolution, batch normalization, and Sigmoid-weighted Linear Unit (SiLU), as shown in Figure 3.

**Figure 4:**
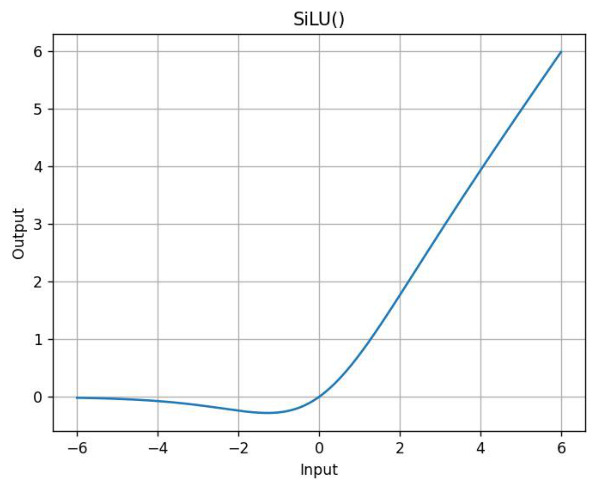
The SiLU activation function.

The CBS primarily serves the following functions. It performs convolution operations by sliding different convolution kernels over the input feature map, automatically extracting local features such as textures and more complex semantic features from gear defect image data. This is the fundamental operation for neural networks to perceive data features. Batch normalization is used to normalize the data for each batch. By using the SiLU activation function, nonlinear factors are introduced. The SiLU activation function is shown in Figure 4. The result of the linear transformation is mapped nonlinearly, enabling the neural network to learn and represent complex nonlinear relationships. This enhances the model’s expressive power.

**Figure 5:**
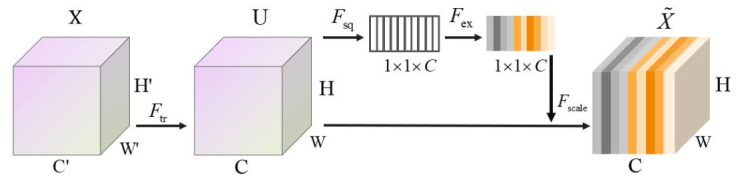
The flowchart of the SE algorithm.

Figure 5 is the flowchart of the SE algorithm. First, the input feature map X is given and transformed by *F*_tr_ to obtain the feature map U. Then, *F*_sq_ performs global average pooling to generate a 1 × 1 × C vector, ensuring that each channel can be represented by a single numerical value. It is shown in Equation (1).

(1) $$z_{c}=F_{sq}(u_{c})=\frac{1}{H\times W}\sum_{i=1}^{H}\sum_{j=1}^{W}u_{c}(i,j)$$

*F*_ex_ is implemented by two fully connected layers. It generates the required weight information through weight W. The fully connected layers W_1_ and W_2_ process the vector *z* to obtain channel weight values *s*. Different values in *s* represent the weight information of different channels, allowing different weights to be assigned to the channels, as shown in Equation (2).

(2) $$s=F_{ex}(z,W)=\sigma (g(z,W))=\sigma (W_{2}(\delta (W_{1}z)))$$

Finally, the module generates the weight vector *s*, assigns weights to the feature map U, and obtains the desired feature map ${\tilde{X}}_{c}$, which has the same size as U. The calculation is as shown in Equation (3).

(3) $${\tilde{X}}_{c}=F_{\mathrm{scale}}(u_{c},s_{c})=s_{c}u_{c}$$

**Figure 6:**
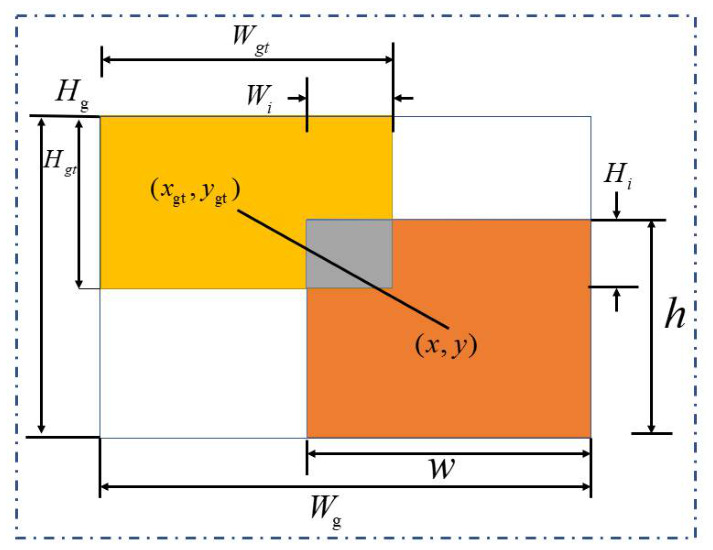
The schematic diagram of the loss function parameters.

The CIoU is used as the loss function to compute localization loss. However, due to its static mechanism, CIoU cannot accurately evaluate the detection performance of small targets with significant differences in width and height. Additionally, CIoU may generate large gradients when dealing with low-quality anchor boxes, thereby slowing down the convergence rate. Therefore, Wise-IoU (WIoU) [43] is used to replace the original model’s CIoU. WIoU has three versions, namely, WIoUv1, WIoUv2, and WIoUv3. WIoUv1 builds an attention-based bounding box loss by increasing the penalty on distance measurement based on DIoU. WIoUv2 and WIoUv3 are obtained by incorporating gradient gains into the focusing mechanism based on WIoUv1. WIoU v3 adopts a bounding box loss function and overcomes the limitations of the monotonic focusing mechanism in WIoU v2 by introducing a dynamic non-monotonic focusing mechanism. Figure 6 illustrates the schematic diagram of the calculation of various parameters in the WIoU loss. (*x*, *y*) presents the center coordinate of the predicted box. (*x*_gt_, *y*_gt_) presents the center coordinate of the true box.

The loss calculation of *L*_WIoUv1_ is divided into two parts, *L*_IoU_ and *R*_WIoU_. *L*_WIoUv1_ represents the bounding box loss of WIoUv1. IoU is the basic bounding box loss. *L*_IoU_ is the basic loss. It measures the overlap value between the predicted and ground truth boxes, as shown in Equation (4). *R*_WIoU_ calculates the ratio of the distance between the center points of the target and predicted box to the length of the diagonal of the minimum enclosing rectangle, as shown in Equation (5). The calculation method of *L*_WIoUv1_ is shown in Equation (6).

(4) $$L_{\mathrm{IoU}}=1-\mathrm{IoU}=1-\frac{W_{i}H_{i}}{wh+w_{\mathrm{gt}}h_{\mathrm{gt}}-W_{i}H_{i}}$$

(5) $$R_{\mathrm{WIoU}}=\exp\left(\frac{{\left(x-x_{\mathrm{gt}}\right)}^{2}+{\left(y-y_{\mathrm{gt}}\right)}^{2}}{{\left(W_{g}^{2}+H_{g}^{2}\right)}^{*s}}\right)$$

(6) $$L_{\mathrm{WIoUv}1}=R_{\mathrm{WIoU}}L_{\mathrm{IoU}}$$

Here, *W_g_* and *H_g_* represent dimensions of the minimum enclosing box. $W_{g}^{2}+H_{g}^{2}$ represents the diagonal length of the minimum enclosing box, and the upper right corner *s indicates the separation operation. It prevents the convergence gradient from being obstructed by *R*_WIoU_.

The calculation method of *L*_WIoUv2_ is shown in Equation (7).

(7) $$L_{\mathrm{WIoUv}2}={\left(\frac{L_{\mathrm{IoU}}^{*}}{\bar{L_{\mathrm{IoU}}}}\right)}^{\gamma }L_{\mathrm{WIoUv}1}$$

Here, $L_{\mathrm{IoU}}^{*}$ represents a monotonic focusing coefficient. $\bar{L_{\mathrm{IoU}}}$ presents the mean value. It serves as a normalization factor in the formula, maintaining a high level of gradient gain and preventing slow convergence in later stages.

The calculation formula for the *L*_WIoUv2_ is shown in Equations (8) and (9).

(8) $$\beta =\frac{L_{\mathrm{IoU}}^{*}}{\bar{L_{\mathrm{IoU}}}}\in [0,+\infty )$$

(9) $$L_{\mathrm{WIoUv}3}=rL_{\mathrm{WIoUv}1},\;r=\frac{\beta }{\delta \alpha^{\beta -a}}$$

Here, *β* is the outlier degree, δ and α are the hyperparameters, and *r* is the gradient gain.

The advantage of choosing the WIoUv3 loss function lies in its ability to dynamically adjust for different detection tasks and targets. At the same time, the WIoUv3 loss function effectively improves the effectiveness and stability of small object detection tasks, thereby enabling the model to exhibit better detection performance.

Aiming at small-scale targets such as pits and extreme aspect ratio targets such as scratches, WIoU adopts the non-monotonic focusing coefficients and dynamic gradient gain mechanisms, respectively.

For small-scale pits, the overlap degree IoU between the predicted box and the real box in the early stage of training is relatively low. WIoU automatically identifies these difficult samples and reduces their loss weights. The model thus avoids being dominated by extremely difficult samples in the training direction, and instead, it prioritizes learning larger and easily recognizable defects, such as obvious scratches or large pits. As the training progresses, the model’s localization accuracy for moderately difficult samples improves, and the quality of the predicted boxes for pits also improves, resulting in a gradual increase in IoU. At this point, WIoU gradually increases its attention to these samples, prompting the model to finely learn the boundaries and positions of small pits.

For scratches with extreme aspect ratios, WIoU does not explicitly introduce aspect ratio penalty terms but indirectly adapts to shape by optimizing normalized distance and non-monotonic focusing. For slender targets, the positioning error in the short side direction is given higher weight. The distance penalty term includes the ratio of center point offset to bounding box size, which enables the model to accurately align the centerline and ends of the scratch.

## 4. Experiments and Results

### 4.1. Datasets and Evaluation Indicators

Gear is one of the core transmission components in mechanical equipment. Its core function is to transmit power and motion accurately through the continuous meshing of gear teeth. Gear defect detection is a key link to ensure transmission accuracy and operation reliability. Through on-site photography and Internet retrieval, a gear defect dataset is constructed.

This dataset contains a total of 948 images and 1828 annotation boxes. The specific distribution of the three types is as follows: 166 images with 368 annotation boxes for rustiness type, 347 images with 619 annotation boxes for intact type, and 482 images with 841 annotation boxes for defect type. The total proportion of the three types of instances is about 20%:34%:46%. Due to the limited size of the dataset, it was only divided into a training set of 750 and a validation set of 198, and no independent test set was established from the original dataset. When evaluating the performance of the model in the future, gear images need to be re-taken for testing. The validation set and training set are strictly non-overlapping, with no image overlap. All image inputs are uniformly scaled to 640 × 640 pixels. The LabelImg tool is used for labeling. Three typical states are selected: intact gears, rusted gears, and defective gears with defects such as missing teeth.

**Figure 7:**
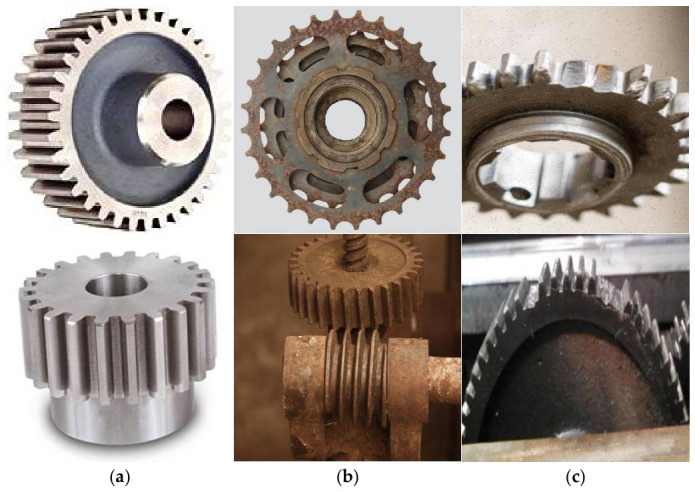
A sample dataset. (**a**) Intact gear; (**b**) Rusted gear; (**c**) Defective gear.

Intact gears, also known as normal gears, refer to gears that are in a design standard state and capable of performing their transmission function normally. The gears do not exhibit failure states such as corrosion or missing teeth. The gear transmission runs smoothly, and the meshing state is good. The system has an excellent lubrication effect, and there is no abnormal temperature rise throughout the entire process. Intact gears are the foundation for the healthy operation of mechanical equipment. Through scientific maintenance, their intact state can be maintained to the greatest extent possible, avoiding losses caused by failures such as corrosion, wear, or even missing teeth. *F*_ex_ refer to the phenomenon where the surface of the gear is damaged due to chemical or electrochemical reactions between the gear surface and surrounding media, such as oxygen and moisture. Rusting can cause the smooth tooth surface to become rough, increase the friction coefficient, and reduce transmission efficiency. After rust particles fall off, they become abrasives, exacerbating the wear and tear on the tooth surface. The pits formed by rusting can become stress concentration points, which are prone to causing fatigue cracks under alternating loads. At the same time, the volume expansion of rust caused by severe corrosion may lead to the disappearance of gear mesh clearance, ultimately resulting in seizure. It leads to immediate equipment downtime and may even trigger safety accidents. Defective gears are one of the most dangerous forms of gear failure. The main causes of gear fracture include overload, fatigue fracture, heat treatment defects, and assembly and installation errors. Overload mainly refers to the sudden start-up or braking of equipment, which causes the gear teeth to instantly bear impact forces far exceeding the design limit, resulting in fracture. Fatigue fracture occurs when the gear tooth root is subjected to alternating stress under normal load. As the number of cycles increase, cracks gradually expand, ultimately leading to tooth fracture. Heat treatment defects refer to the gear being quenched too hard, causing the material to become brittle and generating microscopic cracks that are not detected. These cracks expand during use, eventually leading to tooth fracture. Assembly and installation errors refer to non-parallelism of gear axes and bearing wear, which cause abnormal gear meshing. The force is concentrated on one end of the tooth, resulting in the eccentric load and tooth fracture. Figure 7 shows the images of intact gears, rusty gears, and defective gears.

(1) Accuracy

Accuracy is used to measure the overall accuracy of the model’s predictions. It ranges from 0 to 1, and when the value is close to 1, it indicates that the model has excellent classification performance.

(10) $$Accuracy=\frac{TP+TN}{TP+TN+FP+FN}$$

(2) Precision

Precision refers to the proportion of samples that actually belong to a specific category among all samples predicted as belonging to that category by the model.

(11) $$Precision=\frac{TP}{TP+FP}$$

(3) Recall

Recall is used to calculate the proportion of actual positive samples correctly identified by the model. This indicator represents the coverage of positive samples by the model.

(12) $$Recall=\frac{TP}{TP+FN}$$

(4) F1-Score

F1-Score is used to evaluate model performance by balancing the relationship between the two. It can reflect the model’s performance in accurately identifying positive classes and covering all positive classes.

(13) $$F_{1-score}=2\frac{\left(P\times R\right)}{P+R}$$

(5) Frames Per Second (FPS)

FPS represents the number of images that a model can process within one second. It is an important performance metric for calculating the inference speed of the model.

(14) $$FPS=\frac{\mathrm{Total}\;\mathrm{processing}\;\mathrm{time}\;(/s)}{\mathrm{Total}\;\mathrm{number}\;\mathrm{of}\;\mathrm{images}\;\mathrm{processed}}$$

(6) Parameters

The number of parameters refers to the total count of all trainable parameters in a model, which directly determines the complexity and memory footprint of the model. The larger the number of parameters, the stronger the model’s expressive ability is.

(7) mean Average Precision (mAP)

mAP is used to measure the detection performance of the model. It is calculated by averaging precision, reflecting the overall performance in both classification and localization. mAP@0.5 refers to the mean average precision obtained by calculating the average precision for all categories under the condition of a fixed IoU threshold of 0.5. mAP@0.5:0.95 refers to calculating mAP at multiple different IoU thresholds (usually ranging from 0.5 to 0.95). Then, the arithmetic mean of these mAP values is used as an indicator for comprehensive evaluation of the model’s detection performance.

(8) Confusion Matrix

The confusion matrix is used to display the correspondence between model prediction results and real labels in matrix form. It is possible to analyze the number of correct classifications and the specific situation of incorrect classifications in each category by using the confusion matrix.

### 4.2. Implementation Details

**Figure 8:**
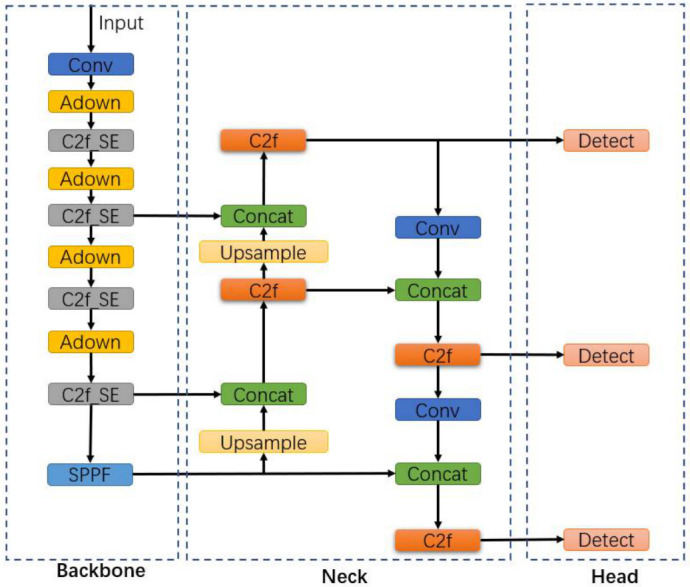
ASW-YOLO network structure.

Based on the original YOLOv8n model, an ASW-YOLO object detection model is proposed. Figure 8 shows the ASW-YOLO network structure. The original YOLOv8n model has problems with high computational complexity and slow convergence speed in industrial defect detection applications. To solve these issues, the backbone network structure needs to be optimized by replacing the convolution module (Conv) with the ADown module. This replacement can reduce the number of parameters and computational complexity while maintaining feature extraction capability.

The original YOLOv8n model has insufficient perception ability for multi-scale defects, especially for detecting small fuzzy defects such as tooth root fractures. To enhance the model’s attention to important features, the SE attention mechanism is introduced in the detection layer. This mechanism enhances the response to defect areas and suppresses noise interference by adaptively recalibrating feature channel weights, thereby improving the perception performance -for multi-scale defect detection. -Meanwhile, the CIoU in the loss function is replaced by WIoU. The WIoU loss function can better handle low-quality samples and positioning errors by dynamically adjusting the weights of bounding box regression.

Table 1 presents the experimental environment and version information. During the training process, the input image resolution was set to 640 × 640, the batch size was 16, and the initial learning rate was 0.01. The SGD optimizer is used for 50 rounds of training.

During training, the Mosaic data augmentation method is used. It randomly selects four images in each training round and concatenates them into a single composite image. The spliced image contains multiple original scenes, simulating the distribution of targets in complex environments and increasing the diversity of training samples. Through this synthesis method, the model can access targets of different scales, including small-sized targets and occluded targets. In addition, Mosaic enhancement reduces the over-fitting tendency of the model to specific training samples by mixing backgrounds and targets from different images, making the model more stable in unknown scenarios. During the training process, Mosaic reinforcement continuously acts on the preceding rounds. But in the last ten rounds of training, the enhancement operation is turned off. The purpose of disabling Mosaic is to gradually adapt the model to the natural distribution of the original image, avoiding feature shift caused by long-term dependence on synthesized images. In the final stage, using raw images without stitching for fine-tuning helps the model regress to the true data distribution, thereby obtaining more reliable detection results on the validation and testing sets. The adjustment of data augmentation strategies aims to balance sample diversity and feature authenticity, ultimately improving the detection accuracy and robustness.

### 4.3. Training Results and Discussion

**Figure 9:**
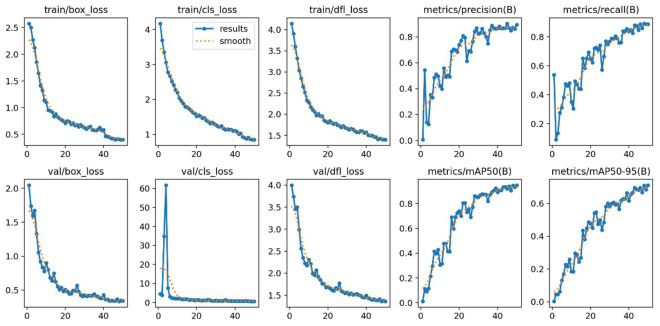
Results of the training.

To evaluate the convergence characteristics and detection performance of the improved ASW-YOLO model, the curve of the training results is obtained, as shown in Figure 9. The six figures on the left side show the changing trends of bounding box regression loss, classification loss, and distributed focus loss on the training and validation sets in sequence. The four figures on the right show the changes in the precision, recall, and mAP of the model on the validation set over training epochs.

The bounding box regression loss shows a decreasing trend in the early stages of training and gradually converges to a stable range. The loss curves of the training set and validation set show a similar trend without significant deviation, indicating that the model’s bounding box localization ability has been continuously optimized during the training process, and no over-fitting has occurred. The distributed focus loss curve also exhibits similar characteristics, with a large initial decrease and stable fluctuations in the later stage, indicating that the model has good stability in fine prediction of bounding boxes.

From the analysis of the evaluation index curve, the precision of the model rapidly increases in the early stages of training and remains high after reaching 0.9. The fluctuation amplitude of the curve is small, indicating that the model has high prediction accuracy and stable performance for positive samples. In the initial stage of training, the model’s recall rate is at a low level. While the training process progresses, the recall rate gradually increases and eventually stabilizes above 0.9. This result indicates that the model significantly enhances the coverage ability of gear surface defect samples and effectively reduces the missed detection rate.

The trend of the comprehensive loss curve and evaluation indicators shows that the ASW-YOLO model exhibits good convergence characteristics during the training process. As all loss functions can steadily decrease, the precision and recall indicators improve synchronously. Moreover, there is no performance degradation in the later stage of training. The detection ability has been effectively enhanced, and the boundary box positioning accuracy and classification accuracy have reached a balance, verifying the effectiveness of the improved scheme. Therefore, the ASW-YOLO has good convergence speed in gear defect detection tasks and can meet the requirements of detection accuracy.

**Figure 10:**
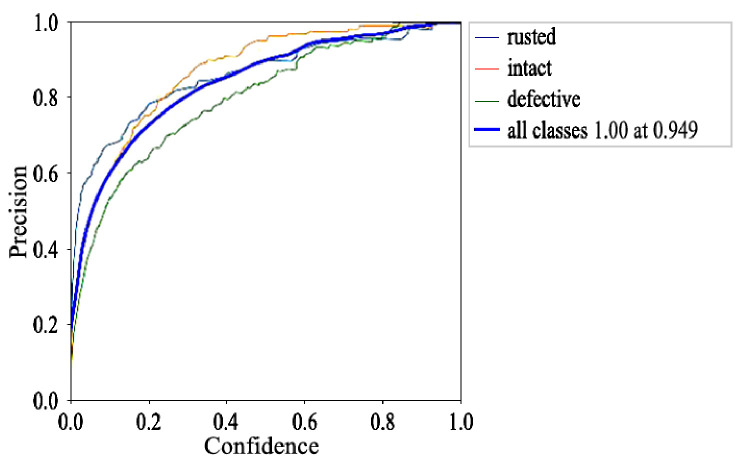
Precision–confidence curve.

Figure 10 is the precision confidence curves of the ASW-YOLO model on the validation set. This curve demonstrates the predictive precision of the model at different confidence thresholds, providing a quantitative basis for the selection of decision thresholds. From the overall detection performance analysis, the accuracy confidence curve of all categories shows a monotonic upward trend in the threshold range from 0 to 1. The AUC reaches 0.949, indicating that the model can maintain high prediction precision within most confidence intervals. As the confidence threshold increases, the model filters out low-confidence predictions, reduces the number of false positives, and the accuracy value gradually approaches 1. This trend conforms to the basic rule that confidence in object detection tasks is positively correlated with predictive reliability.

Rusted gears have a faster rate of precision increase in the confidence threshold range below 0.2, but the precision growth rate slows down in the medium- to high-confidence range of 0.2 to 0.8. The curve trend of intact gear is close to the average curve of the entire category, and the accuracy steadily improves within the confidence interval of 0.2 to 0.6. When the threshold exceeds 0.8, its accuracy value basically coincides with the curve of the entire category, indicating that the model’s recognition accuracy of the intact gear is consistent with the overall detection performance. Defective gears have significantly higher precision values than the other two categories within the low-confidence interval, with a confidence threshold below 0.3. This feature indicates that the model has a more significant response to the characteristics of defective gears and can distinguish them from other categories under lower-confidence conditions, which has practical significance for the need to prioritize defect detection in industrial inspection.

**Figure 11:**
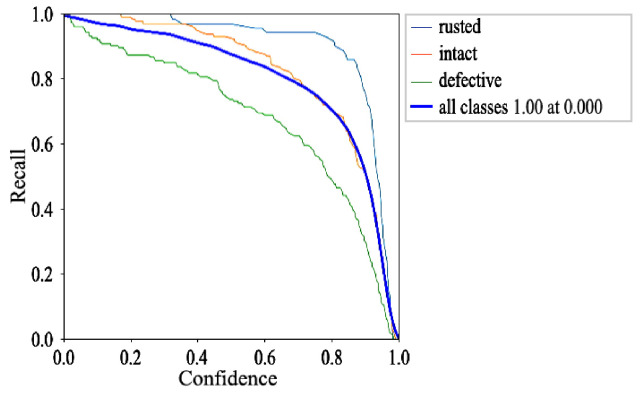
Recall–confidence curve.

Figure 11 is the recall confidence curve. This curve reflects the detection ability of the model for various types of targets under different confidence threshold conditions, providing a quantitative basis for evaluating the risk of missed detections and detection coverage.

The recall confidence curve for all categories reaches 1.00, indicating that all real targets can be detected by the model without confidence constraints. The recall rate shows a continuous downward trend. In the high-confidence interval (threshold greater than 0.8), the rate of decline significantly accelerates, and when the confidence approaches 1.0, the recall rate drops to 0. This change pattern is consistent with the basic relationship between confidence and recall in object detection tasks, that is, increasing the confidence threshold can reduce false positives but, at the same time, increase the probability of missed detections.

The curve of rusted gears is at a relatively high level within the full confidence interval, and the rate of decline is relatively gentle. During the process of increasing the confidence threshold from 0 to 0.8, the decrease in recall rate is relatively small, indicating that the model has a low risk of missed detection of rusted gears and can maintain a high detection rate even under high confidence constraints. The curve trend of intact gears is closer to the average curve of the entire category. Its recall rate remains at a high level, indicating that the model’s ability to recognize intact gears is stable and maintains a low rate of missed detections. Among the three types of gears, the recall curve of defective gears is the worst. The rate of decline is significantly faster than that of intact gears and rusted gears in the medium- to high-confidence interval. When the confidence threshold exceeds 0.3, the decrease in the recall rate of defective gears significantly increases, indicating that the model’s ability to detect defective gears is more sensitive to the confidence threshold.

**Figure 12:**
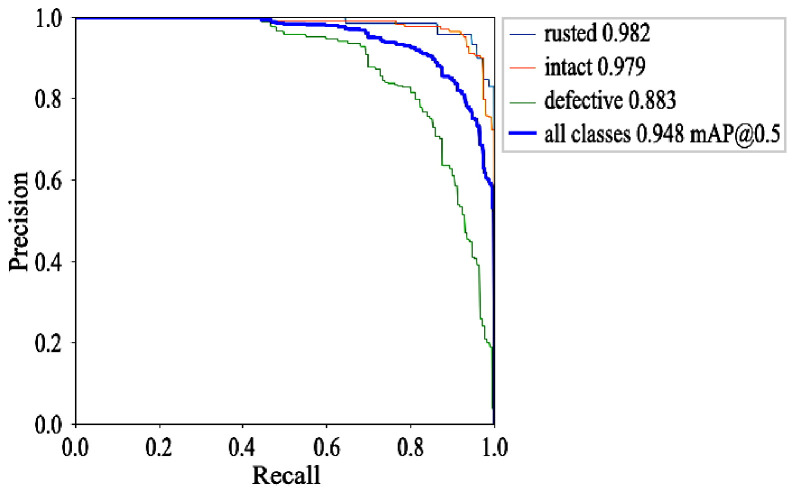
The precision–recall curve.

Figure 12 is the precision–recall curve. It reflects the trade-off between precision and recall rate of the model under different decision thresholds. The area under the precision–recall curve for all classes reaches 0.948. The curve trend indicates that the model can maintain stable accuracy while maintaining a high recall rate. The trade-off between the two is relatively balanced. In the interval where the recall rate gradually increases from 0 to 0.9, the decrease in the precision value is relatively small. Hence, the model has the ability to distinguish positive and negative samples.

The value of rusted gears is 0.982, which is the highest among the three categories. The detection of corroded gears by the model requires a certain precision cost in pursuit of high recall, but it still maintains good overall performance. The value of the intact gear is 0.979, and the curve maintains precision above 0.95 within the range of recall increasing from 0 to 0.95, with a gentle downward trend. This indicates that the model has sufficient feature learning for intact gears and can maintain high accuracy within a wide recall range. The value of defective gears is 0.883, which is relatively low among the three categories. Its curve is close to rusted gears in the range where the recall rate is below 0.8. When the recall rate exceeds 0.8, the rate of decrease in precision significantly accelerates. The model has demonstrated overall good detection performance in gear defect detection tasks.

**Figure 13:**
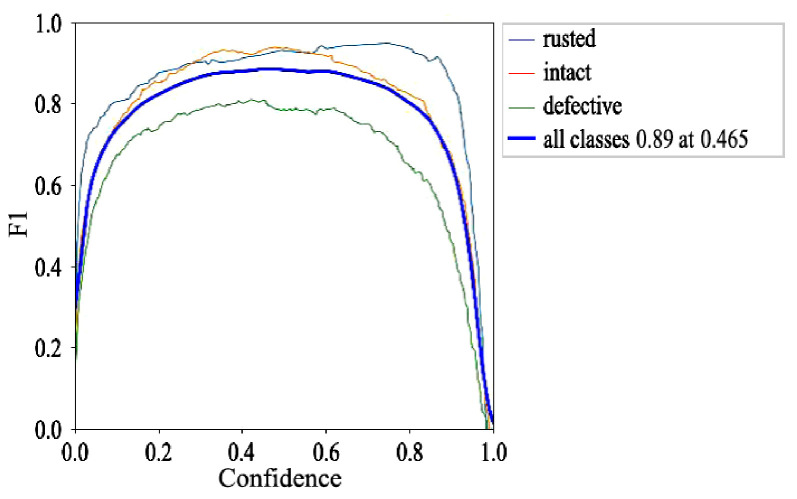
The F1–confidence curve.

Figure 13 is the F1–confidence curve. It provides a quantitative reference for determining the optimal decision threshold in practical applications. The value of F1 is 0.89, and the confidence threshold is 0.465. The F1 curve of rusted gears remains above 0.8 within the confidence interval of 0.3 to 0.8, with a relatively flat curve shape and small fluctuations. This feature indicates that the model can maintain stable comprehensive performance in identifying rusted gears within a wide range of confidence, with low sensitivity to threshold selection. The curve trend of intact gears is highly consistent with the average curve of all categories, and the F1 value steadily increases within the confidence interval of 0.2 to 0.5. When the threshold approaches 0.8, its F1 value basically coincides with the curve of the entire category. The recognition ability of the model for intact gears is synchronized with the overall detection performance, and this category shows stable performance in balancing accuracy and recall. The F1 curve of defective gears exhibits different characteristics. In the low threshold range with a confidence level below 0.2, the curve rises slowly, and its value is lower than other categories during the same period. Throughout the entire range of confidence, defective gears are located below the average curve.

**Figure 14:**
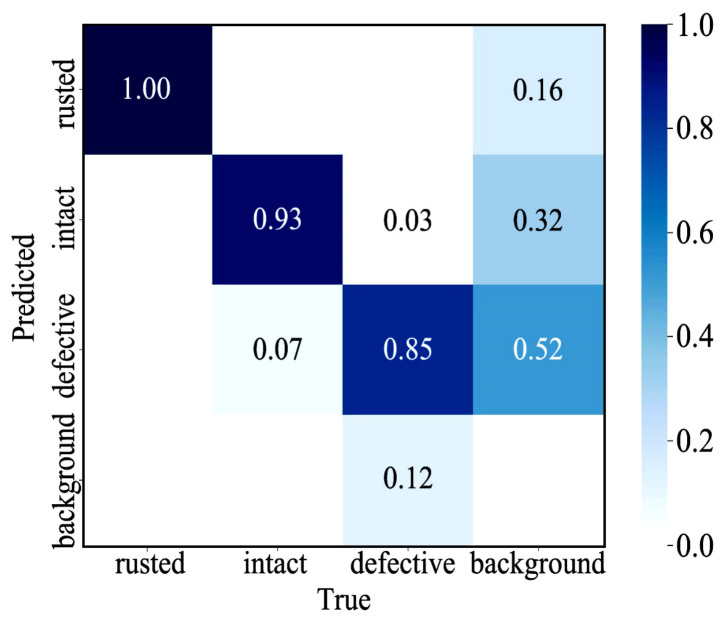
A normalized confusion matrix.

Figure 14 is the normalized confusion matrix. The classification accuracy of rusted gears reaches 1.00, and all samples within the true category of rusted gears are correctly identified, with no samples misclassified as other categories or backgrounds. This result indicates that the model has sufficient feature learning for rusted gears, and the feature extraction module can effectively capture discriminative information. Meanwhile, the model can accurately distinguish rusted gears from other categories and backgrounds.

The classification accuracy of intact gears is 0.93. The overall classification reliability of this category is high, but there are still a small number of samples that are confused with defective gears and backgrounds. The misjudgment between intact gears and defective gears may be due to the visual similarity between some defective samples and intact gears or the small defect area and unclear features. The classification accuracy of defective gears is 0.85. It is the lowest among the three categories, with the highest proportion of misjudgments, reflecting the difficulty of detection in this category. The main reasons for this confusion are as follows. The definition of intact categories is not precise enough. -These categories actually include several regions that are visually similar to defective categories, such as gear textures, oil stains, etc. The training did not introduce difficult negative sample mining, and there is a lack of reinforcement learning for easily confused non-defect areas, such as edge highlights and slight scratches.

Based on the above analysis, the ASW-YOLO model exhibits differentiated classification performance in three types of gear detection tasks. The detection accuracy of rusted gears is optimal, and the classification accuracy reaches the ideal level. The intact gears have good detection performance and a low misclassification rate. The detection performance of defective gears is relatively weak, with a high rate of misclassification, mainly focused on confusion with intact gears and the background.

### 4.4. Comparison Experiment

To verify the effectiveness of the ASW-YOLO model, relevant experiments are conducted under the same PyCharm 2024.3.5 (Community Edition) deep learning framework, as shown in Table 2. Each model was trained with multiple comparison methods in the same hardware and software environment to ensure fairness and reproducibility of the comparison results. All models adopt a unified training parameter and dataset partitioning method and record various evaluation indicators after completing training. By analyzing the experimental results, the performance of the ASW-YOLO model in gear defect detection tasks and its advantages compared to comparative methods can be systematically evaluated.

(1) Calculation efficiency (GFLOPs). Experimental data show that YOLOv11n has the lowest computational complexity among the comparison models, but its detection accuracy correspondingly decreases. The original YOLOv8n showed a good balance between accuracy and computational complexity in the YOLO series models, so it is selected as the baseline model for improvement. The ASW-YOLO model maintained high detection accuracy at moderate computational levels, with the mAP@0.5 of 0.948 and mAP@0.5:0.95 of 0.7113. The results verify the advantages of the improved scheme in terms of computational resource utilization efficiency.
(2) Parameter (M). The parameter size of the ASW-YOLO model proposed in this article is 2.73 M, which is 9.30% lower than the original YOLOv8n model’s 3.01 M. Compared with YOLO11n (2.59 M), ASW-YOLO has a slightly increased parameter count, but the accuracy improvement is more significant, and the performance benefits brought by parameter growth are considerable. Compared with Faster R-CNN and SSD, ASW-YOLO has the advantage of parameter quantity and is more suitable for deployment in resource-constrained industrial detection equipment.
(3) mAP@0.5. The mAP@0.5 of YOLOv7 and YOLOv11n are significantly higher than those of other compared models, reflecting their comprehensive advantages in target localization and classification tasks. The SSD model performs well in object detection with smaller input sizes, but its mAP@0.5 is still lower than that of the YOLO series models. Faster R-CNN uses a region recommendation network to generate candidate regions, which can more accurately locate gear defects and reduce missed and false detections. Its mAP@0.5 is better than SSD but slightly lower than the YOLO series. The mAP@0.5 of ASW-YOLO is 0.948, which is the highest one.
(4) mAP@0.5:0.95. The mAP@0.5:0.95 of ASW-YOLO is 0.7113, which is also the highest one. YOLOv11n and YOLOv8n followed closely behind with 0.672 and 0.6685, respectively. The mAP@0.5:0.95 of Faster R-CNN and SSD are 0.634 and 0.629, respectively, which are within an acceptable range but have been surpassed by the new generation of detection architectures. The above data indicate that the ASW-YOLO model effectively improves its feature extraction and representation capabilities through backbone network replacement, attention mechanism introduction, and loss function optimization, providing a feasible solution for high-precision object detection in resource-constrained environments.
(5) FPS. The ASW-YOLO model has a lower detection speed than YOLOv11n. Specifically, ASW-YOLO achieved an accuracy improvement of over 7% while sacrificing approximately 25% of inference speed. For defect detection tasks, improving accuracy often has a higher priority. Compared with YOLOv8n (74.78 FPS) and SSD (77.28 FPS), ASW-YOLO achieves significant leadership in mAP@0.5 and mAP@0.5:0.95 while maintaining comparable or even faster inference speed, demonstrating good overall performance.

At the same time, the lightweight detector EdgeYOLO is selected as the control for the comparative experiment. Both run on the same CPU platform and ONNX Runtime inference backend. The inference speed of ASW-YOLO is 81.73 FPS, while EdgeYOLO is 82.4 FPS, which is basically the same. But the mAP@0.5 and mAP@0.5:0.95 of ASW-YOLO are 37.5% and 62.3% higher than EdgeYOLO, respectively. The computational complexity has decreased by 72%, and the parameter count has decreased by 53%. The above results indicate that ASW-YOLO improves detection accuracy and computational efficiency while maintaining real-time detection capability.

### 4.5. Ablation Experiment

To evaluate the effectiveness of various improvement modules, ablation experiments were completed in the same environment based on the YOLOv8n baseline. The experiment is based on the original YOLOv8n, and it gradually introduces C2f_SE attention module, ADown downsampling module, and the WIoU loss function. The experiment uses different module combination configurations for comparison and verification. The experimental data are collected in the same dataset and environment to ensure comparable results. Table 3 is the result of the ablation experiment.

The original YOLOv8n model achieved the mAP@0.5 of 90.3%, the mAP@0.5:0.95 of 66.85%, the precision of 85.7%, and the recall of 82.5% on the test set. It indicates that the YOLOv8n architecture has basic effectiveness in object detection tasks, but there is still room for improvement for complex scenarios.

C2f_SE module: After introducing C2f_SE attention module on the baseline model, mAP@0.5 increased to 90.6%, precision increased to 87.8%, recall slightly increased to 82.7%, and mAP@0.5:0.95 increased to 67.93%. This module adjusts the weights of different channel features adaptively, making the model more focused on feature regions related to gear defects, suppressing background noise interference, and thus improving feature expression ability. It is consistent with the SENet method proposed by Hu et al. [42], which suggests that channel attention mechanisms can effectively enhance feature representation, but when used alone, the improvement in localization accuracy is limited, possibly due to the module’s role in shallow features.

ADown module: After replacing the Conv with ADown downsampling, the mAP@0.5 of the model significantly increased to 92.7%, the recall rate increased to 87.4%, and the precision slightly decreased to 84.5%. The ADown module dynamically adjusts the downsampling rate through learnable parameters, which reduces the resolution of feature maps while preserving more fine-grained information. Compared with traditional stride convolution, it can reduce information loss. The experimental results show that this module has a significant effect on improving the recall rate. The decrease in precision may indicate the introduction of some false detection, which needs to be balanced with other modules.

A quantitative comparative experiment is designed to verify the retention ability of the ADown module for defect-related pixels. Two models are used for gear defect detection in the experiment. One is the original YOLOv8n model. The other type is the YOLOv8n model that only replaces the original downsampling module with ADown convolution, denoted as ADown-YOLO. The retention effect of dual-path pooling on defective pixels is evaluated through three indicators. The activation defect IoU is used to quantify the localization accuracy of YOLO models for defect locations. The higher the IoU, the more focused the model’s attention is on the true defect area, rather than the background or irrelevant textures. It also proves that the model has an accurate positioning ability and can indirectly reduce the risk of false detection of the background as defects. A higher background suppression ratio is better. An increase in this value indicates that the model has less false activation of the background region. Higher activation confidence correlation is better. The increase in this value indicates a stronger consistency between the model’s response to defective pixels and the final detection confidence, i.e., higher feature sensitivity. The experimental results are shown in Table 4.

Compared to the YOLOv8n baseline, the activation defect IoU of ADown-YOLO decreased slightly from 0.2034 to 0.1985. The background suppression ratio increased from 0.5121 to 0.5292. The activation confidence correlation coefficient increased from 0.2685 to 0.4183, with a relative increase of 55.8%. The above indicators comprehensively indicate that the ADown module indeed retains more pixels related to defects through the dual-path pool structure while effectively suppressing background noise, thereby enhancing the sensitivity of features to defects. At the same time, the activation defect IoU of ASW-YOLO reached 0.2536, an increase of 24.7% compared to YOLOv8n’s 0.2034. The background suppression ratio is 0.4514, lower than YOLOv8n’s 0.5121, corresponding to a 11.8% decrease in background interference. The activation confidence correlation coefficient is 0.4596, much higher than YOLOv8n’s 0.2685, with an improvement of 71.1%. The above results demonstrate that the attention module of ASW-YOLO can more accurately focus on defect areas and suppress background noise.

WIoU loss function: After replacing the original CIoU with WIoU, the mAP@0.5 of the model has increased to 93.1%, the recall rate has increased to 89.3%, and the mAP@0.5:0.95 has reached 70.37%, but the precision has decreased to 82.7%. WIoU dynamically adjusts the weights of bounding box regression to give fair optimization strength to targets of different sizes, especially for small targets such as tooth root fracture defects, which have better adaptability.

The influence of module combination: The experiment further investigated the synergistic effect of WIoU with ADown and C2f_SE. When WIoU is used in combination with ADown (No. 6), mAP@0.5 reaches 93.9%, mAP@0.5:0.95 reaches 70.30%, the recall rate is 87.3%, the precision is 85.0%, and the performance is better than using each module separately, indicating that ADown downsampling optimization and WIoU scale adaptive loss can complement each other. The combination of WIoU and C2f_SE (No. 5) achieved mAP@0.5 of 92.2%, mAP@0.5:0.95 of 69.03%, the recall rate of 87.4%, and the precision of 87.2%. Although slightly lower than the combination with ADown, the precision remained at a high level, indicating that the attention mechanism helps suppress false positives.

Complete model performance: After integrating C2f_SE, ADown, and WIoU into YOLOv8n, the model achieved the best performance. The mAP@0.5 reached 94.8%, an improvement of 4.5% from the baseline. The mAP@0.5:0.95 reached 71.13%. The recall rate was 88.6%, and the precision was 89.3%. The above data indicate that the three improved modules have good complementarity in gear defect detection tasks. ADown enhances fine-grained feature preservation, C2f_SE guides the model to focus on key areas, and WIoU optimizes the regression accuracy of targets at different scales.

### 4.6. Visual Analysis and Detection Prediction

**Figure 15:**
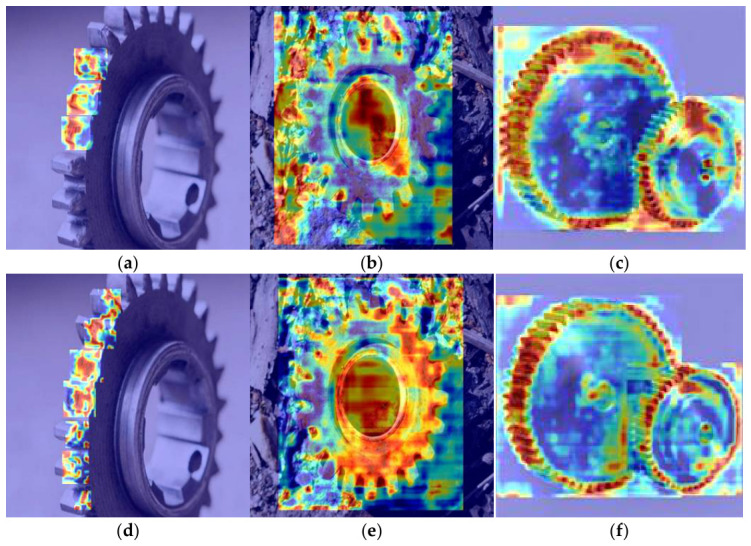
YOLOv8n and ASW-YOLO heat map comparison. (**a**) YOLOv8n model—Defective gear; (**b**) YOLOv8n model—Rusted gear; (**c**) YOLOv8n model—Intact gear; (**d**) ASW-YOLO model—Defective gear; (**e**) ASW-YOLO model—Rusted gear; (**f**) ASW-YOLO model—Intact gear.

The Grad CAM is used to visualize the feature extraction process before and after the model improvement, as shown in Figure 15. The heat maps before improvement are shown in Figure 15a–c, and the heat maps after improvement are shown in Figure 15d–f. The red and yellow regions represent the feature areas that the model focuses on during classification or localization, which contribute significantly to the final prediction results. The blue and purple areas indicate low model attention and minimal impact on decision-making. By comparing the differences in the heat map before and after improvement, the defect areas can be qualitatively analyzed.

Three types of samples were selected for the experiment and input into YOLOv8n and ASW-YOLO, respectively. Grad CAM was used to generate corresponding heat maps. As shown in the figure, the thermal response area of the corroded gear sample in the improved model is relatively scattered, with some highlighted areas deviating from the actual corroded position. There is a certain degree of false activation in the background area, indicating that the model was affected by noise interference during feature extraction and failed to focus on key defect areas fully. The heat map of intact gear samples is concentrated in the gear body, but the heat map in the edge area is weak, which may affect the judgment of integrity. In the defective gear samples, the heat map covers some of the defect positions, but there is insufficient response intensity and regional discontinuity, reflecting the limited feature capture ability of the model for small defects.

The heat map of the improved model shows significant changes. In the defective gear samples, the heat map shows a high-intensity continuous response at the defect location, especially for the precise localization of small defects such as tooth root fractures. The response area boundary is clear and distinguishable from the background. For rusted gear samples, the high response area is more concentrated in the rusted area, highly overlapping with the real defect area, and the background interference is significantly suppressed. The heat map of intact gear samples is concentrated on the overall contour of the gear, and the response in the edge area is enhanced, indicating that the model has a more comprehensive grasp of the structural characteristics of intact gears. The improved model provides more accurate feature representation and enhanced localization ability for various gear defects, providing a visual basis for reliable detection in industrial scenarios.

**Figure 16:**
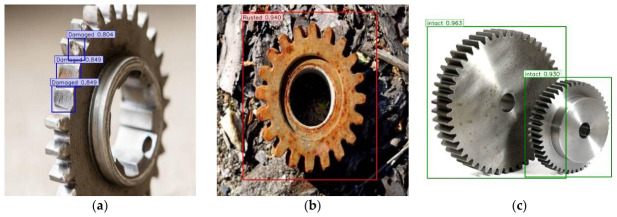
Results of detection prediction. (**a**) Defective gear; (**b**) Rusted gear; (**c**) Intact gear.

To verify the performance of the improved model in actual detection tasks, randomly selected gear samples are predicted on the test set, and the confidence score of the model output is recorded, as shown in Figure 16. The predicted confidence levels of defective gear samples are 0.849 and 0.804, respectively. The predicted confidence level of the rusted gear sample is 0.940, indicating that the model has a high level of reliability in discriminating this category. The predicted confidence levels of intact gear samples were 0.963 and 0.930, respectively. Both types of samples obtained high-confidence outputs, reflecting the stable and reliable feature recognition of intact gears by the model. The distribution of confidence in the prediction of the test set provides a reference for subsequent model threshold selection and application deployment.

**Figure 17:**
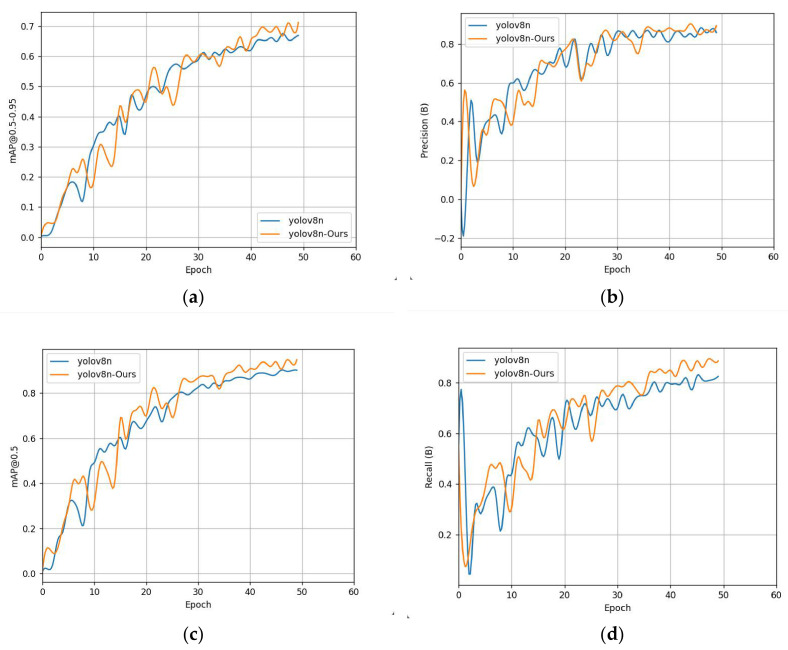
Comparison of training results between YOLOv8 and ASW-YOLO. (**a**) mAP@0.5:0.95; (**b**) Precision; (**c**) mAP@0.5; (**d**) Recall.

On the basis of completing ablation experiments and testing set validation, in order to analyze the performance evolution characteristics, the key indicator changes of original YOLOv8n and ASW-YOLO under the same training parameters were compared. The analysis results are shown in Figure 17.

Figure 17a is the training curve of mAP@0.5:0.95. It shows a gradual upward trend with increasing training epochs. The curve consistently remains above the YOLOv8n model. At last, the curve is around 0.71, while the corresponding index of the YOLOv8n model is about 0.67. Figure 17b is the precision curve. The precision of both types of models shows a fluctuating upward trend and a large fluctuation amplitude in the early stages of training. The precision of the ASW-YOLO model first shows a rapid decline, and then, it recovers, while that of the YOLOv8n model gradually increases. Subsequently, the accuracy growth rate of both types of models slows down and gradually stabilizes. Throughout the entire training process, the accuracy curve of the ASW-YOLO remained higher than that of the YOLOv8n model in most rounds. Especially in the later stages of training, the accuracy of ASW-YOLO remained stable at around 0.89, while the corresponding index of the YOLOv8n model was about 0.86. The improved model achieved an effective improvement in accuracy. Figure 17c is the curve of mAP@0.5. The curve of ASW-YOLO is consistently higher than that of YOLOv8n in most rounds. Especially in the later stages of training, the mAP@0.5 is at around 0.95, while the corresponding index of the YOLOv8n model is about 0.90. Figure 17d is the curve of recall. The recall rate of ASW-YOLO first drops rapidly and then rises, while the recall rate of YOLOv8n gradually increases. Subsequently, the recall rate growth rate of both types of models slows down and gradually stabilizes. The recall curve of ASW-YOLO was consistently higher than that of YOLOv8n in most rounds. The recall of ASW-YOLO remained stable at around 0.89, while the corresponding index of the YOLOv8n was about 0.83. The improved model achieved an effective improvement in the recall rate. This result indicates that targeted optimization of the YOLOv8n model can reduce the missed detection rate.

### 4.7. Generalization and Robustness Experiment

In order to comprehensively evaluate the generalization ability of ASW-YOLO in industrial environments, three lighting variants (strong highlight, striped shadow, and non-uniform light) and two metal texture variants (directional texture and rough texture) are generated through image processing, totaling seven variant test sets. The ASW-YOLO is used for inference and calculation, separately for mAP@0.5 and mAP@0.5:0.95. The experimental results are shown in Table 5.

The model exhibits strong robustness to common lighting fluctuations such as strong highlights, stripe shadow, and non-uniform light. The decrease in mAP@0.5 is less than 2%. There are changes in metal material for directional textures and rough texture; the decrease in mAP@0.5 is only 5.5% to 6.8%, significantly better than expected. This indicates that the depth features extracted by ASW-YOLO have good invariance to uneven lighting and common metal surface processing traces and can meet the needs of most gear quality inspection production lines with stable lighting and clean environments.

In order to evaluate the robustness of the model to common disturbances at industrial sites, two degradation validation sets, motion blur and lens contamination, are constructed. The motion blur validation set simulates the trailing effect caused by production line vibration or object movement by applying linear motion blur with random direction and a kernel size of 5~21 to the original clear image. The lens contamination validation set randomly overlays semi-transparent circular dark spots with a coverage area of 5% to 15% on the image, simulating real pollution such as dust, oil stains, and water mist. During the generation process, only the pixel content is changed, keeping the original annotation box unchanged to ensure that degradation does not affect the true position and size of defects. Both degradation sets contain the same number of images and corresponding labels as the original validation set.

YOLOv8n and ASW-YOLO are used as comparison objects. Inference is conducted on three validation sets: clean, motion blur, and lens contamination, using a uniform input size of 640 × 640. mAP@50 and mAP@0.5:0.95 indicators are recorded, and the results are shown in Table 6.

Under motion blur interference, the absolute performance of the two models is very close. The mAP@50 of ASW-YOLO is 85.3%, and YOLOv8n is 85.2%. However, in terms of performance degradation from clean images to motion blur, YOLOv8n decreased from 90.3% to 85.2% (a decrease of 5.1%), while ASW-YOLO decreased from 94.8% to 85.3% (a decrease of 9.5%). The reason for the decrease is that the attention mechanism introduced in ASW-YOLO is more sensitive to high-frequency edge details, and motion blur destroys this fine-grained information, resulting in a more significant relative decrease. Nevertheless, both have mAP@50 exceeding 85% under motion blur, still meeting the application requirements of most real-time quality inspection scenarios.

When lens contamination is introduced, the performance of ASW-YOLO is completely unaffected. mAP@50 is 94.8%, while YOLOv8n decreased from 90.3% to 89.7%. This indicates that ASW-YOLO has excellent robustness against lens surface contamination, suitable for industrial quality inspection environments where lenses are prone to dirt and stains.

Based on three sets of experiments, it can be concluded that ASW-YOLO has significantly better accuracy on clean images. Under the interference of lens contamination, its performance is not affected and exhibits a certain degree of robustness. Under motion blur, although the decrease is slightly greater than YOLOv8n, the absolute accuracy remains 85.3%. Therefore, this model is also applicable to gear quality inspection scenarios where the lens is susceptible to contamination and motion blur is controllable.

## 5. Conclusions

The ASW-YOLO model is proposed to solve the problems of multi-scale defect omission and difficulty in balancing model lightweight and accuracy in gear defect detection. This model introduces the C2f_SE channel attention mechanism, which enhances the responsiveness to important features. The WIoU is used to improve the regression accuracy for targets of different scales. The traditional downsampling convolution is replaced with ADown to optimize information preservation during the feature compression process.

(1) Lightweight and computational efficiency. The ASW-YOLO model achieved the mAP@0.5 of 94.8%, recall of 88.6%, and the precision of 89.3% on the gear test set, with a computational complexity of 7.5 GFLOPs and the parameter count of 2.73 M. Compared with the original YOLOv8n, the parameters are decreased by 9.30%, and the computational complexity is decreased by 8.54%. This result validates the ADown module’s ability to retain effective information during feature compression.
(2) Multi-scale defect detection capability. By combining the dynamic calibration of channel features with the C2f_SE module, the detection precision of the model for different shapes and sizes has been improved. The ablation experiment data showed that after introducing ADown and C2f_SE, mAP@0.5 increased by 3.7% compared to baseline.
(3) Module synergy effect. The ablation experiments show that the combined gain of the ADown module, C2f_SE attention mechanism, and WIoU loss function is better than that of a single module. After integration of the three, mAP@0.5 reached 94.8%. mAP@0.5:0.95 reached 71.13%. The recall rate and accuracy rate reached 88.6% and 89.3%, respectively.
(4) Applicability to industrial scenarios. ASW-YOLO maintains real-time inference speed (81.73 FPS) and a significantly higher mAP@0.5 (94.8%) than Faster R-CNN (85.4%) and SSD (84.9%), and both parameter and computational complexity are at a lower level. In complex industrial environments, the model has high stability in detecting three types of gears.

Future work can be carried out in the following directions. Firstly, using hyperparameter search methods, including Bayesian optimization and genetic algorithm, to optimize parameters such as channel attention weight, downsampling, and step size, and explore their optimal compatibility with gear defect features. Secondly, by combining semi-supervised learning strategies and utilizing unlabeled samples to expand training data, the bottleneck problem of small sample defect categories can be alleviated. Thirdly, ASW-YOLO will be used to detect surface defects in lightweight components. By using the distribution location, type, and severity of defects as feedback constraints, the boundary conditions and stress threshold settings for topology optimization are fed back to form a complete closed loop.

## Figures and Tables

**Table 1:** Experimental environment and version information.

Experimental Environment	Version Information
Operation system	Windows 11
CPU	12th Gen Intel Core i7-12700H
GPU	NVIDIA GeForce RTX 3060 Laptop 6 GB
PyTorch	1.12.1
CUDA	11.3
Driver	566.41

**Table 2:** Comparison of different models.

Model	Input	Backbone	Calculation (GFLOPs)	Parameter (M)	mAP@0.5	mAP@0.5:0.95	FPS
Faster-RCNN	600 × 600	ResNet50	370.2 G	137.099 M	0.854	0.634	11.4
SSD	300 × 300	VGG16	62.7 G	26.285 M	0.849	0.629	77.28
EdgeYOLO	640 × 640	ELAN-Darknet	27.23 G	5.8 M	0.689	0.438	82.4
YOLOv7	640 × 640	E-ELAN	106.5 G	37.6 M	0.826	0.645	30.097
YOLOv11n	640 × 640	CSPDarknet	6.4 G	2.59 M	0.874	0.672	116.89
YOLOv8n	640 × 640	CSPDarknet	8.2 G	3.01 M	0.903	0.6685	74.78
ASW-YOLO	640 × 640	ASESPDarknet	7.5 G	2.73 M	0.948	0.7113	81.73

**Table 3:** Results of the ablation experiment.

No.	YOLOV8n	C2f_SE	ADown	WIOU	mAP@0.5 (%)	mAP@0.5:0.95 (%)	Recall (%)	Pr (%)
1	√				90.3	66.85	82.5	85.7
2	√	√			90.6	67.93	82.7	87.8
3	√		√		92.7	69.32	87.4	84.5
4	√			√	93.1	70.37	89.3	82.7
5	√	√		√	92.2	69.03	87.4	87.2
6	√		√	√	93.9	70.30	87.3	85.0
7	√	√	√		94.0	69.67	88.2	85.4
8	√	√	√	√	94.8	71.13	88.6	89.3

**Table 4:** The model feature evaluation index.

Model	Activation Defect IoU	Background Suppression Ratio	Activation Confidence Correlation
YOLOv8n	0.2034	0.5121	0.2685
ADown-YOLO	0.1985	0.5292	0.4183
ASW-YOLO	0.2536	0.4514	0.4596

**Table 5:** Detection performance of ASW-YOLO under various light and texture distortions.

	mAP@0.5	Rate of Change	mAP@0.5:0.95	Rate of Change
ASW-YOLO	0.9466	–	0.7123	–
Strong highlight	0.9422	−0.5%	0.7076	−0.7%
Striped shadow	0.9346	−1.3%	0.7087	−0.5%
Non-uniform light	0.9473	+0.1%	0.7113	−0.1%
Directional texture	0.8944	−5.5%	0.6588	−7.5%
Rough texture	0.8822	−6.8%	0.6377	−10.5%

**Table 6:** Comparison of detection performance between different models under motion blur and lens contamination.

	Condition	mAP@50	Rate of Change	mAP@0.5:0.95	Rate of Change
YOLOv8n	Clean	90.3	–	69.0	–
	Motion blur	85.2	−5.1	62.8	−6.2
	Lens contamination	89.7	−0.6	66.4	−2.6
ASW-YOLO	Clean	94.8	-	71.1	-
	Motion blur	85.3	−9.5	62.1	−9
	Lens contamination	94.8	−0	70.9	−0.2

## Data Availability

The original contributions presented in this study are included in the article. Further inquiries can be directed to the corresponding author.
